# Ischemic Central Retinal Vein Occlusion Accompanied by Macular Edema: A Case Report

**DOI:** 10.7759/cureus.66152

**Published:** 2024-08-05

**Authors:** Yuga Pawar, Sachin Daigavane, Archana R Thool, Uma Swaminathan, Zoya Javed

**Affiliations:** 1 Ophthalmology, Jawaharlal Nehru Medical College, Datta Meghe Institute of Higher Education and Research, Wardha, IND

**Keywords:** crvo, central retinal vein occlusion, intravitreal bevacizumab, hyperviscosity syndrome, diabetes

## Abstract

One of the primary reasons for discernible or complete loss of sight in middle-aged and older persons is central retinal vein occlusion (CRVO). Three intravitreal bevacizumab (Avastin) injections were administered to a 39-year-old male patient as part of his therapy for his prior diabetic retinopathy due to uncontrolled diabetes. The patient was advised to undergo gonioscopy and an undilated iris examination to look for angle neovascularization during the next three months of follow-up. We demonstrated an extraordinary instance of unilateral ischemia CRVO (macular edema), where the main cause of risk was insulin resistance. To avoid a situation like this, close observation and diabetes management are recommended.

## Introduction

In the middle-aged and elderly population, central retinal vein occlusion (CRVO) [1] is a significant cause of marked or complete vision loss. Vein occlusion in another eye may develop in 6-17% of cases [2]. CRVO is the primary cause of blindness, besides diabetic retinopathy [3]. Hypertension, diabetes mellitus, hyperlipidemia, homocysteinemia, glaucoma, and other conditions are the main contributing factors.

Venous stagnation is caused by thrombosis inside the major retinal vein, which causes disc enlargement, extensive cotton wool spots, pre-retinal bleeding, and nerve fiber layer damage. This spectacular fundus is sometimes called “the blood and thunder” fundus. Population-based studies indicate that between 0.1 and 0.7% of people have CRVO [4]. According to a population-based study, the frequency for those aged 65 and above was 1.3%, while the cumulative incidence across 15 years was 0.5%. Hyperviscosity, coagulopathy, migraine, and atherosclerotic risk factors (age, diabetes, and hypertension) are linked to central retinal vein blockage or thrombosis. Retinal vein obstruction usually has an unclear etiology [5].

Vision loss often starts subacutely, but it can occasionally become severe, as opposed to the sudden loss of vision associated with central retinal artery occlusion. Severe venous stasis can cause a halt in retinal blood circulation on the artery side, which can lead to an infarction. A Marcus gun pupil is frequently observed in this situation [4]. Here, we present a captivating example of unilateral ischemic CRVO.

## Case presentation

A 39-year-old patient came to the hospital complaining of decreased and blurry vision in his left eye. The loss of vision had started abruptly, increased gradually, and was painless. There was no redness, floaters, or swelling in the eyes. He had uncontrolled diabetes mellitus in the past. He was taking metformin, but, currently, he was taking a medication that combined 500 mg of metformin with 1 mg of glimepiride. His overall examination was normal, and his vital signs were stable.

The results of an ophthalmologic test showed that the eye on the left side had an estimated visual acuity of 6/60 and that of the right eye was 6/6. The response of the left eye’s pupil revealed a sluggishly reacting pupil, while the left eye’s swinging light reflex revealed a Marcus gun pupil. In comparison to the left eye, which had an intraocular pressure of 19 mmHg, the right eye had a pressure of just 17 mmHg. The right eye’s optic disc was healthy; however, a fundus analysis of the left eye revealed a 0.4 cup-disc ratio and hyperemic disc with blurred disc margins.

**Figure 1:**
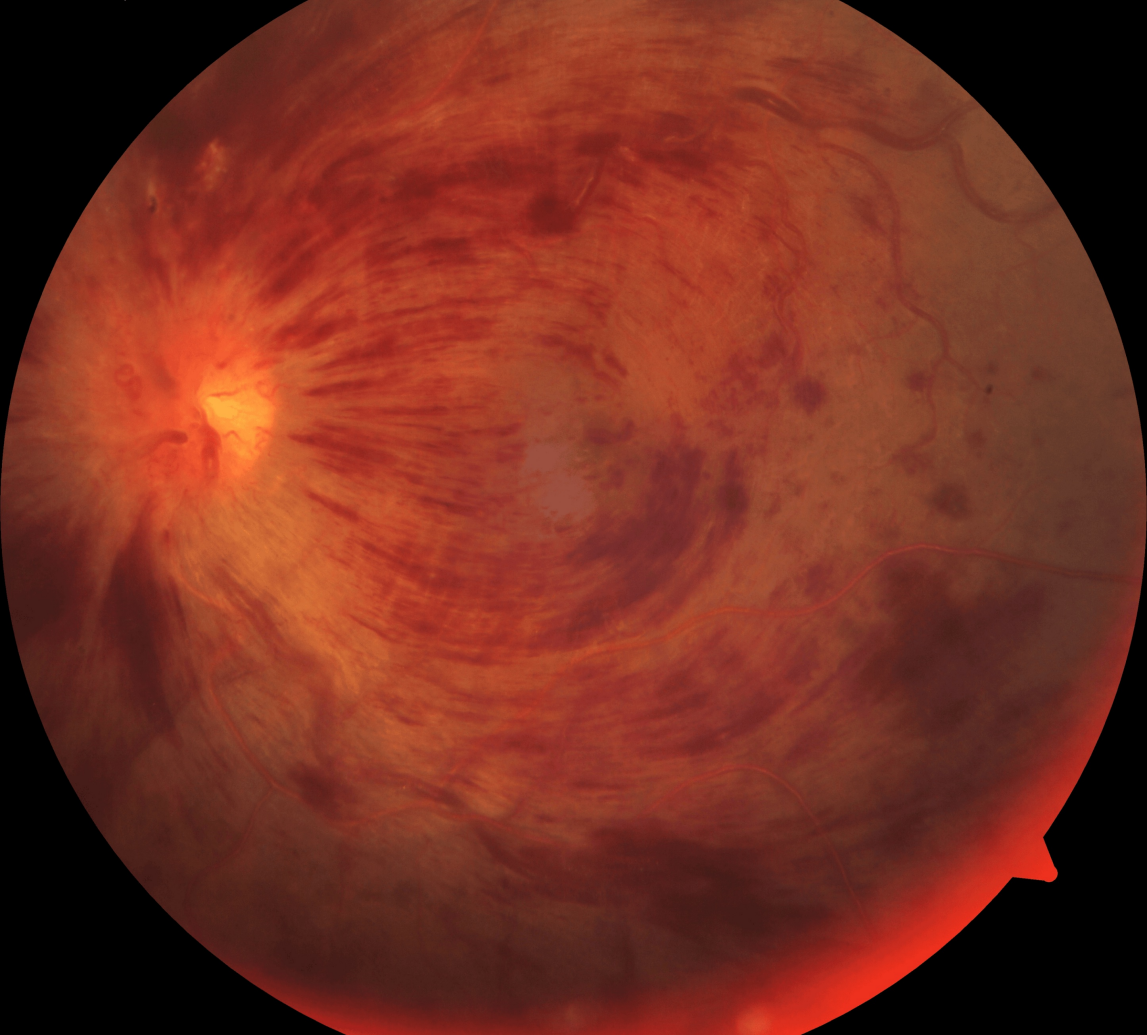
A colored fundus image of the left eye demonstrating tortuous veins around the disc exhibiting many flame-shaped, dot-blot, and intraretinal hemorrhages in all four retinal quadrants and disc edema.

A colored fundus image of the left eye demonstrated tortuous veins around the disc that exhibited many flame-shaped, dot-blot, and intraretinal hemorrhages in all four retinal quadrants and disc edema (Figure 1).

**Figure 2:**
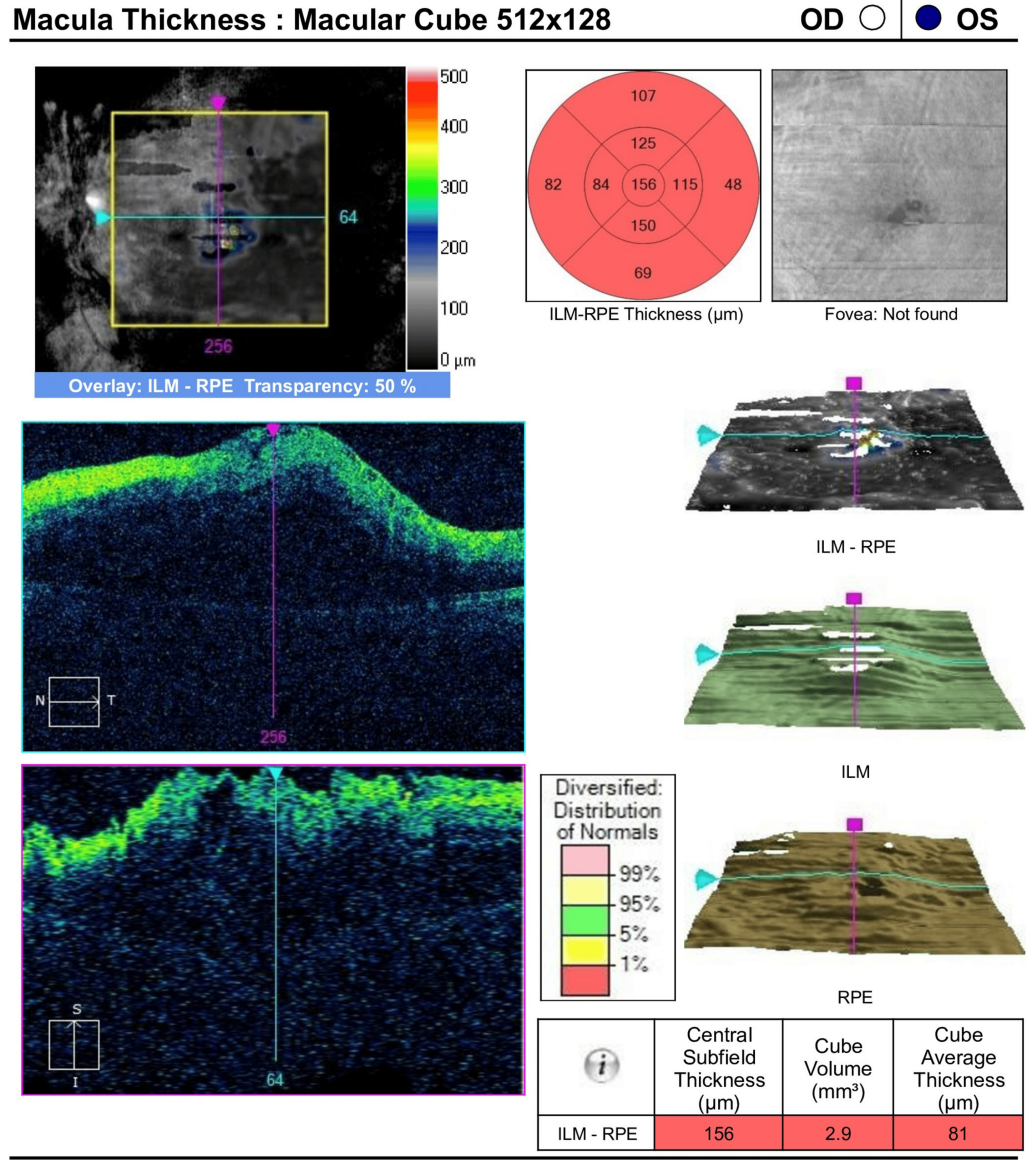
Optical coherence tomography of the left eye to quantify the macular thickness in this case, indicating macular edema.

Optical coherence tomography was used to quantify the macular thickness in this case, indicating macular edema (Figure 2).

The patient’s laboratory test results are displayed in Table 1.

**Table 1:** Patient’s laboratory test results.

Lab parameters	Observed value	Normal range
Hemoglobin	14	13–17 g%
White cell count	2,600	4,000–11,000/mm^3^
Platelets	1.96	1.5–4 lakh/mm^3^
Mean corpuscular volume	85	83–101 fL
Urea	24	19–43 mg/dL
Creatinine	1.1	0.66–1.25 mg/dL
Potassium	4.8	3.5–5.1 mmol/L
Sodium	137	137–145 mmol/L
Aspartate aminotransferase	79	Male: <17–59 U/L; Female: 14–56 U/L
Alkaline phosphatase	110	38–126 IU/L
Alanine aminotransferase	39	Male: <50 U/L; Female: <35 U/L
Total protein	7.5	6.3–8.2 g/dL
Total bilirubin	1.1	0.2–1.3 mg/dL
Creatinine kinase myoglobin binding	8	9–16
Troponin-I	1.5	Male: up to 13 pg/dL; Female: up to 9 pg/dL
Serum calcium	8.2	8.4–10.2 mg/dL
Serum magnesium	1.9	1.6–2.3 mg/dL
Serum phosphorous	4.2	2.5–4.5 mg/dL
International normalized ratio	1.2	<1.5
Random blood sugar	189	140–200 mg/dL
Hemoglobin A1C	9.0	<5.7

The diagnosis was made of a diabetic patient with CRVO and macular edema. The differential diagnosis is presented in Table 2. A total of three 0.25 mL intravitreal bevacizumab injections were administered (one per month). After three months, the left eye’s visual acuity improved to 6/9. In addition, brimonidine, timolol, and moxifloxacin were recommended for improving eye vision and halting the spread of bacterial infections. The patient underwent gonioscopy and an undilated iris examination throughout the next three months to look for signs of neovascularization in the iris or disc tissues. At the three-month follow-up, the patient’s sharpness of vision in the left eye was 6/9 (with a pinhole).

**Table 2:** Differential diagnosis.

Differential diagnosis	Supporting signs	Contradicting signs
Radiation retinopathy	Hard exudate, cotton wool spots, macular edema	No exposure to any source of radiation, no vitreous hemorrhage, no neovascularization
Papilledema	Blurred disc margins	No symptoms of papilledema (severe headache) or retinal hemorrhages present all over the fundus
Diabetic retinopathy	Hard exudates, cotton wool spots, macular edema	Tomato splash appearance of the fundus, rapid onset of signs, chronic presentation, no neovascularization
Retinopathy from anemia	Cotton wool spots	No retinal hypoxia, no hypoproteinemia, hemoglobin of 14 g/dL

**Figure 3:**
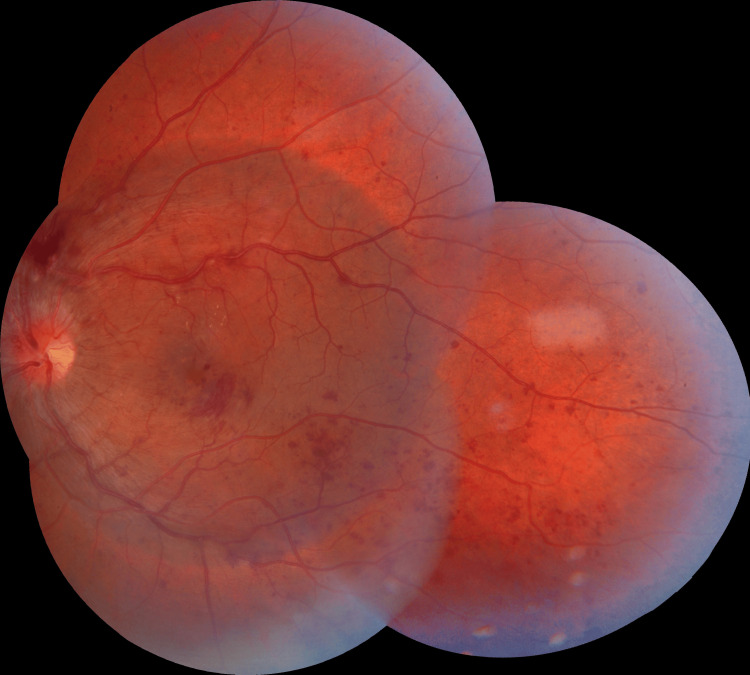
Left eye’s fundus examination after three months showing a disk with a cup-disc ratio of 0.4, blurred disc margins, few flame-shaped hemorrhages, a few dot and blot hemorrhages, and intraretinal hemorrhages in every region (resolving type). In the macular area, a few cotton wool spots can be noted (resolving type).

When the left eye’s fundus was examined after three months, it exposed a disk with a cup-disc ratio of 0.4, blurred disc margins, a few flame-shaped hemorrhages, a few dot and blot hemorrhages, and intraretinal hemorrhages in every region (resolving type). In the macular area, a few cotton wool spots (resolving type) were noted (Figure 3).

**Figure 4:**
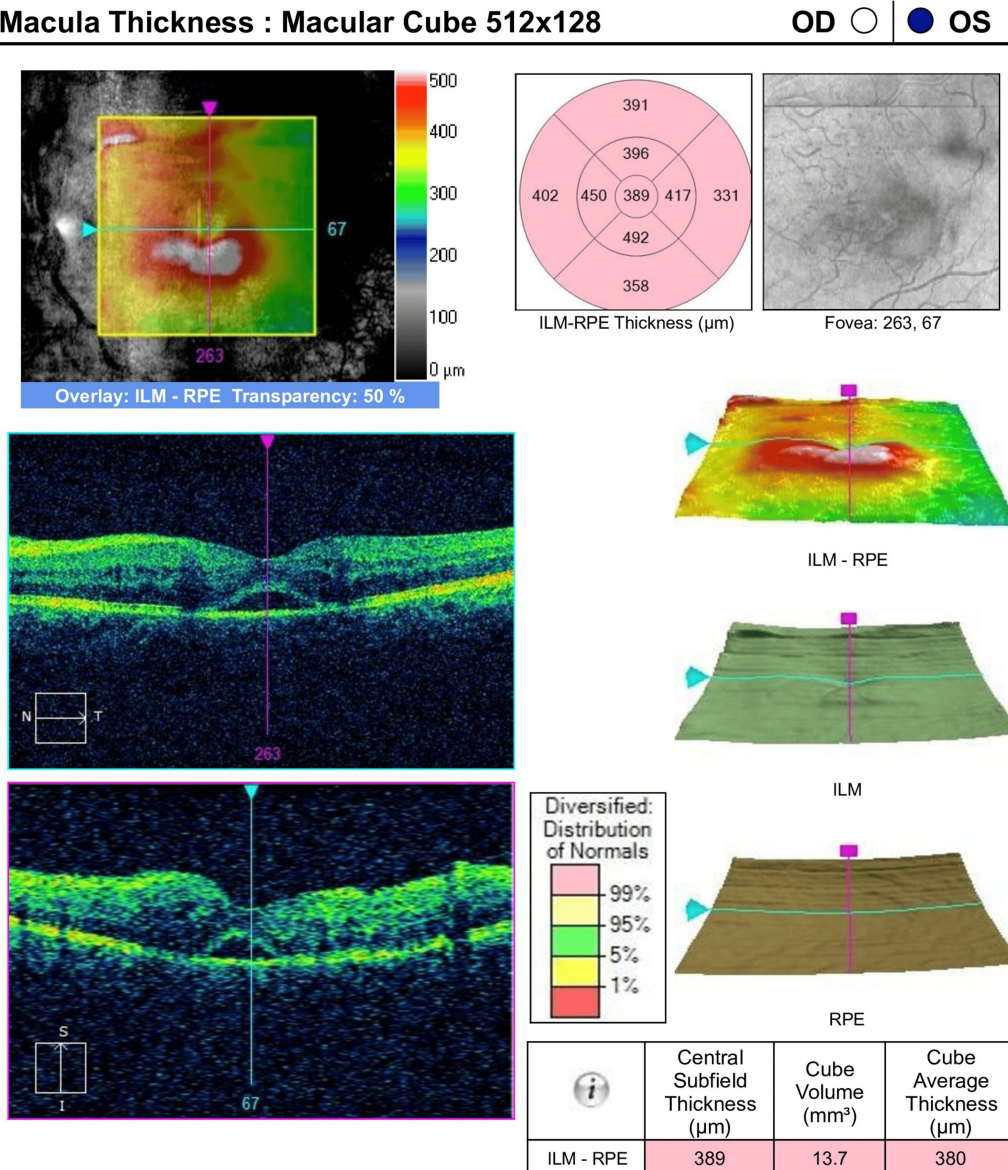
Optical coherence tomography of the left eye after three months revealing resolving macular edema with pigmentary epithelial detachment.

Optical coherence tomography revealed resolving macular edema with pigmentary epithelial detachment (Figure 4).

## Discussion

In ischemic CRVO, the blocked site is the cribriform plate of the ethmoid bone or the region immediately posterior to that. It is defined as more than 10 DD of non-perfusion patients, who are elderly and visually impaired; of these, 60% develop iris neovascularization (INV), up to 33% develop neovascular glaucoma (NVG), and 10% also present with retinal branch occlusion (usually cilioretinal artery due to low perfusion pressure of the choroidal system) [1].

Some individuals with non-ischemia CRVO may develop ischemic symptoms overnight or over time. The majority of persons affected by CRVO are elderly (aged over 50). Insulin resistance, cholesterol buildup, and elevated arterial pressure are important risk factors. Hyphema, hyperviscosity syndromes, sarcoidosis, syphilis, vasculitis, elevated intraocular and orbital pressure, and glaucoma are additional risk factors [6]. Additional variables may have an impact on the natural history of CRVO.

Demographic variables consist of gender and age. Systemic factors include cardiovascular disease and raised blood hematocrit volume. Ophthalmic factors that have been associated with poor functional outcomes include alterations in pigments in the macula, retinal collaterals, formation of the epiretinal membrane in cases with established macular edema, and glaucoma [7]. A previous study found a correlation between retinal vein occlusion and traditional atherosclerotic risk factors [7]. They found that CRVO is mostly associated with diabetes, followed by hypertension and hyperlipidemia.

Reduced eyesight at presentation, presence of a Marcus gun pupil, and hypertension are indicators of risk for the commencement of NVG in an eye with CRVO [8]. Retinal circulation in an ocular CRVO is improved by reducing intraocular pressure. There is a 10% possibility that branch retinal vein occlusion or CRVO will develop in the adjacent eye.

As visual impairment quantifies the degree of non-perfusion revealed on fluorescein angiography, it is a powerful early indication of the beginning of INV/angle neovascularization (ANV) in individuals first diagnosed with CRVO. Pan-retinal laser photocoagulation (PRP) is now the gold standard therapy for INV or ANV, and patients with ischemic CRVO are advised to undergo this procedure regularly.

The preventive PRP recommendation was not supported by the results of the Central Venous Occlusion Study (CVOS). CVOS predicted a lower INV risk with early PRP. That being said, there was no statistically significant decrease [8]. The CVOS encouraged physicians to promptly pursue PRP when INV or ANV occurs and to closely evaluate patients’ eyes (including gonioscopy and iris inspection under an undilated slit lamp) during the first six months of the patient’s treatment. Prompt PRP is more effective in identifying INV and anterior segment neovascularization reversion than selective PRP or photodynamic treatment (PDT). PDT may also be used safely in treatment [9]. Selective PRP or PDT can also be used safely to manage anterior segment neovascularization secondary to ischemic CRVO, despite the superiority of PRP in determining INV and anterior segment neovascularization regression [10].

## Conclusions

This case highlights the peculiar picture of ischemic CRVO, where the predominant risk factor was diabetes, resulting in macular edema and vision impairment. Our main concern was controlling diabetes as a prophylactic to safeguard the other eye. It is crucial to keep a close watch for any indications of INV or ANV in such patients.

## References

[1] Mantha MK, Suvvari TK, Kotipalli LN, Kota T (2021). A classic case of ischemic central retinal vein occlusion with macular edema. MGM J Med Sci.

[2] Yelne P, Mathurkar S, Kumar S (2024). Concomitant two-temporal cilioretinal artery occlusion (CLRAO) with impending central retinal vein occlusion (CRVO) in a young adolescent with protein C deficiency: a rare case. Cureus.

[3] Walinjkar RS, Khadse S, Kumar S, Bawankule S, Acharya S (2019). Platelet indices as a predictor of microvascular complications in type 2 diabetes. Indian J Endocrinol Metab.

[4] (1993). Baseline and early natural history report. The Central Vein Occlusion Study. Arch Ophthalmol.

[5] Alasil T, Lee N, Keane P, Sadda S (2009). Central retinal vein occlusion: a case report and review of the literature. Cases J.

[6] Klein R, Moss SE, Meuer SM, Klein BE (2008). The 15-year cumulative incidence of retinal vein occlusion: the Beaver Dam Eye Study. Arch Ophthalmol.

[7] Bhagat N, Goldberg MF, Gascon P, Bell W, Haberman J, Zarbin MA (1999). Central retinal vein occlusion: review of management. Eur J Ophthalmol.

[8] Pathak A, Gupta S, Kumar S, Agrawal S (2017). Evaluation of cardiovascular autonomic nervous functions in diabetics: study in a rural teaching hospital. J Pract Cardiovasc Sci.

[9] Sharma S, Daigavane S, Shinde P (2024). Innovations in diabetic macular edema management: a comprehensive review of automated quantification and anti-vascular endothelial growth factor intervention. Cureus.

[10] Parodi MB, Friberg TR, Pedio M, Fiotti N, Di Stefano G, Ravalico G (2007). Panretinal photocoagulation and photodynamic therapy for anterior segment neovascularization secondary to ischemic central retinal vein occlusion. Ophthalmic Surg Lasers Imaging.

